# Bank vole genomics links determinate and indeterminate growth of teeth

**DOI:** 10.1186/s12864-024-10901-2

**Published:** 2024-10-30

**Authors:** Zachary T. Calamari, Andrew Song, Emily Cohen, Muspika Akter, Rishi Das Roy, Outi Hallikas, Mona M. Christensen, Pengyang Li, Pauline Marangoni, Jukka Jernvall, Ophir D. Klein

**Affiliations:** 1 Baruch College, City University of New York, One Bernard Baruch Way, New York, NY 10010 USA; 2 The Graduate Center, City University of New York, 365 Fifth Ave, New York, NY 10016 USA; 3 Program in Craniofacial Biology, Department of Orofacial Sciences, University of California, San Francisco, San Francisco, CA 94158 USA; 4 Division of Paleontology, American Museum of Natural History, Central Park West at 79th Street, New York, NY 10024 USA; 5 Cornell University, 616 Thurston Ave, Ithaca, NY 14853 USA; 6 New York University College of Dentistry, 345 E 34th St, New York, NY 10010 USA; 7 Institute of Biotechnology, University of Helsinki, Helsinki, FI-00014 Finland; 8 Department of Pediatrics, Cedars-Sinai Guerin Children’s, 8700 Beverly Blvd., Suite 2416, Los Angeles, CA 90048 USA; 9 Department of Bioengineering, Stanford University, 443 Via Ortega, Rm 119, Stanford, CA 94305 USA; 10 Department of Geosciences and Geography, University of Helsinki, Helsinki, FI-00014 Finland

**Keywords:** Evolution, Selection, Glires, Molar, Root, Dental, Development, Genome, Rodent, Tooth

## Abstract

**Background:**

Continuously growing teeth are an important innovation in mammalian evolution, yet genetic regulation of continuous growth by stem cells remains incompletely understood. Dental stem cells responsible for tooth crown growth are lost at the onset of tooth root formation. Genetic signaling that initiates this loss is difficult to study with the ever-growing incisor and rooted molars of mice, the most common mammalian dental model species, because signals for root formation overlap with signals that pattern tooth size and shape (i.e., cusp patterns). Bank and prairie voles (Cricetidae, Rodentia, Glires) have evolved rooted and unrooted molars while retaining similar size and shape, providing alternative models for studying roots.

**Results:**

We assembled a *de novo* genome of *Myodes glareolus*, a vole with high-crowned, rooted molars, and performed genomic and transcriptomic analyses in a broad phylogenetic context of Glires (rodents and lagomorphs) to assess differential selection and evolution in tooth forming genes. Bulk transcriptomics comparisons of embryonic molar development between bank voles and mice demonstrated overall conservation of gene expression levels, with species-specific differences corresponding to the accelerated and more extensive patterning of the vole molar. We leverage convergent evolution of unrooted molars across the clade to examine changes that may underlie the evolution of unrooted molars. We identified 15 dental genes with changing synteny relationships and six dental genes undergoing positive selection across Glires, two of which were undergoing positive selection in species with unrooted molars, *Dspp* and *Aqp1*. Decreased expression of both genes in prairie voles with unrooted molars compared to bank voles supports the presence of positive selection and may underlie differences in root formation.

**Conclusions:**

Our results support ongoing evolution of dental genes across Glires and identify candidate genes for mechanistic studies of root formation. Comparative research using the bank vole as a model species can reveal the complex evolutionary background of convergent evolution for ever-growing molars.

**Supplementary Information:**

The online version contains supplementary material available at 10.1186/s12864-024-10901-2.

## Background

**Fig. 1:**
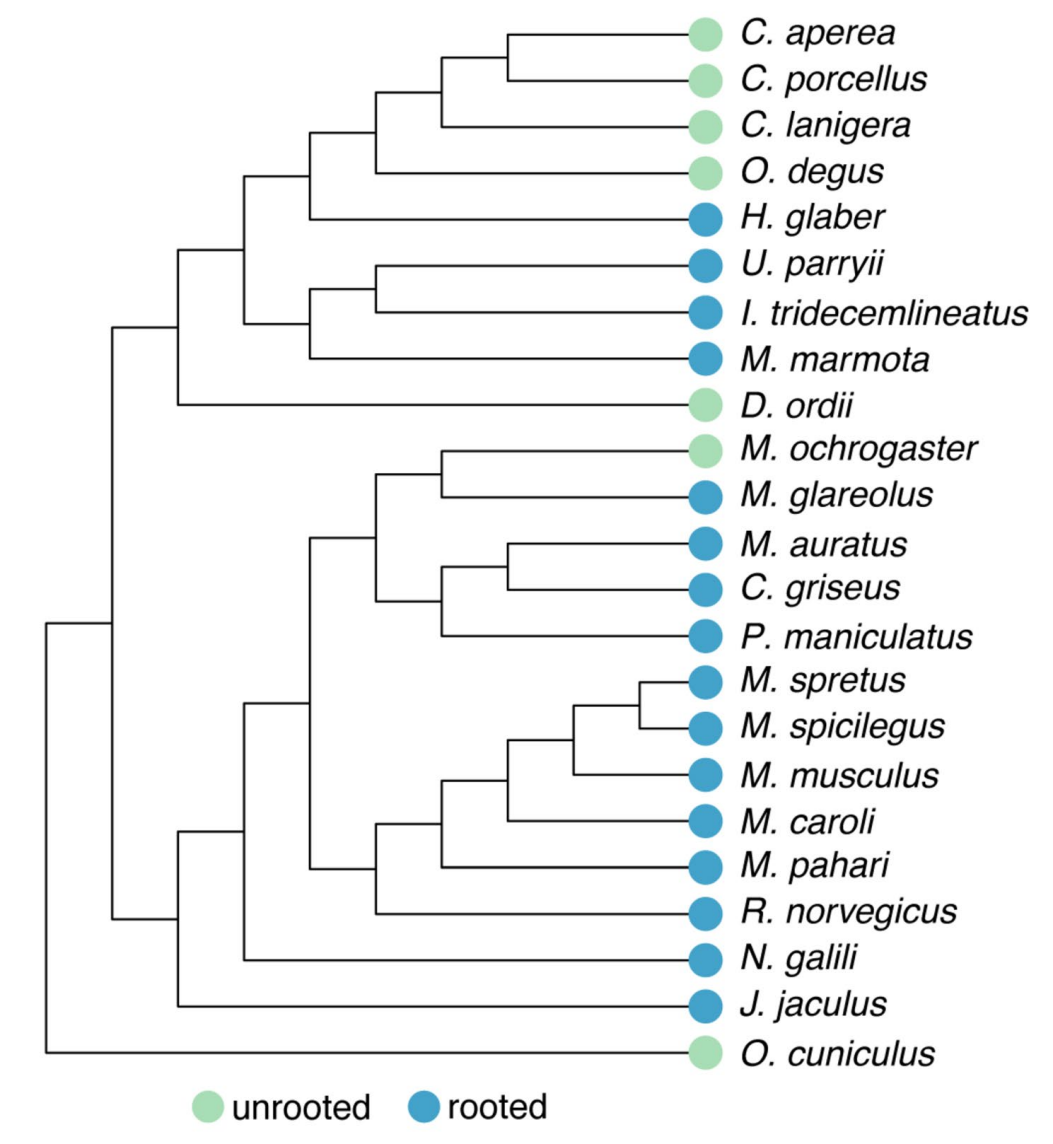
Species tree of Glires based on the Ensembl Compara species tree. Whether each species has rooted or unrooted molars is indicated by colored circles at the tip of each branch. Note that unrooted, or hypselodont, molars have evolved multiple times across Glires. This topology was the basis for orthology analyses

Hypselodonty, or the presence of unrooted and thus continuously-growing teeth, has evolved multiple times in mammals. Glires—the clade containing rodents, rabbits, and their relatives—have hypselodont incisors, and multiple Glires have also convergently evolved hypselodont molars (Fig. 1). At least in rodents, hypselodont molars evolved considerably later than hypsodont molars, which are high crowned but rooted, and both evolved later than hypselodont incisors. In Glires, molars appear to increase in crown height from brachydonty (low-crowned, rooted), through hypsodonty (high-crowned, rooted), toward hypselodonty (high-crowned, unrooted) [2]. Mice (*Mus musculus*), the primary mammalian model species of dental research, have hypselodont incisors but retain brachydont molars. Because of this, mice cannot provide information about the hypsodont teeth that preceded hypselodonty.

Mammalian teeth sit in bony sockets, held in place by soft tissue (periodontal ligament) attached to cementum-covered tooth roots [3]. Originally, ligamentous tooth attachment may have arisen along with a reduction in the rate of tooth replacements, providing greater flexibility for repositioning the teeth as the dentary grows [3, 4]. Consequently, the limited replacement of mammalian teeth (two sets of teeth in most mammals and one in Glires) may have spurred the evolution of hypsodont and hypselodont teeth, both with high crowns that compensate for tooth wear from gritty or phytolith-heavy diets [5, 6], and resulted in further modification of the anchoring roots. The convergent evolution of unrooted molars in Glires presents an opportunity to identify whether consistent developmental and genomic changes underlie the formation of hypselodont teeth in different species, in turn revealing the conserved mechanisms that produce tooth roots. Furthermore, the relatively recent evolution of molar hypselodonty, starting in the Middle Miocene (approximately 16 − 12 Ma) [2], should provide molecular evidence for the steps required to make a continuously growing organ.

Dental development proceeds from the tooth germ, composed of epithelium and mesenchyme, through phases known as the bud, cap, and bell [7]. Multipotent enamel epithelium differentiates into the cells that form the tooth crown [8–11]. As development progresses in rooted teeth, the epithelium at the tooth apex transitions first to a tissue called Hertwig’s epithelial root sheath, and eventually cementum-covered roots [9, 10]. Studies have identified numerous candidate genes and pathways with various roles during root development, such as *Fgf10*, which decreases in expression at the beginning of root formation [12–18]. Although research on mouse molars has identified genetic signals related to root formation, a number of the key genes studied have broad developmental roles, such as *Wnt* family members [14], or overlap considerably with genes also involved in patterning the size and shape of the tooth [17, 19–22]. This overlap between shape and root expression patterns confounds our ability to identify a clear signal initiating root formation.

Evolutionary novelties such as high-crowned hypsodont and hypselodont molars can arise from differences in gene expression and regulation [23–26]. Evolutionarily conserved gene expression levels produce conserved phenotypes, and changes in gene regulatory networks have long been linked to morphological evolution [27, 28]. The order of genes along a chromosome (synteny) can affect gene expression and regulation, as regulatory sequences are often located near their target genes (cis-regulatory elements) [29–31]. Genome rearrangements that place genes near new regulatory elements may change the expression and selective environment of those genes; these small-scale rearrangements of genes may be common in mammals [32–34]. Likewise, regions of chromosomes that form topologically associated domains may experience similar selective pressures, including selection against rearrangement [35, 36]. Genes involved in molar development are not syntenic in the mouse genome nor are genes with organ-specific expression [37], and thus the regulatory or selection effects of co-localization need not apply to all dental genes at once. Changes in genome architecture between Glires species thus may result in different selective and expression environments for dental genes that could result in the evolution of hypselodont molars.

To establish a model rodent species with hypsodont molars, linking brachydont and hypselodont molars, we sequenced and annotated a highly-complete *de novo* genome of *Myodes glareolus*, the bank vole. Although other draft genomes for the bank vole have been published, the only annotated genome publicly available at time of publication has a contig N50 less than 1 million base pairs (GCF_902806735.1), which can affect downstream analyses; our goal was to improve genomic resources available for the species through *de novo* sequencing efforts. The bank vole is increasingly used in medical and environmental research, ranging from studying zoonotic diseases [38] to immune responses [39, 40], and even assessing environmental remediation efforts through heavy metals that accumulate in vole teeth [41, 42], thus our efforts may be of use beyond dental research. The quality of our genome assembly and gene annotation were validated by the comparable patterns of dental gene expression levels between bank voles and the well-studied mice, yet we found key differences related to the more rapid crown formation of vole molars. Therefore, the bank vole’s hypsodont molars bridge the gap between brachydont mice (low-crowned, rooted molars) and hypselodont prairie voles (*Microtus ochrogaster*; high-crowned, unrooted molars), and reduce the effects of morphological differences on measurements of root formation signaling. We performed a suite of genomic and transcriptomic tests of the bank vole genome in a broad phylogenetic context to test the hypothesis that dental genes are undergoing positive selection and exhibit different expression patterns in species with unrooted, hypselodont molars. We predicted that genes without conserved syntenic relationships in these species would be more likely to have sites under positive selection or significantly different expression. Although we identified genes which lacked synteny and genes undergoing positive selection among species with unrooted molars, there was not a clear pattern between synteny and selection. Two genes under positive selection provide strong candidates for future functional analyses of dental development in bank voles and prairie voles to elucidate the genetic basis of tooth root formation.

## Results

### Orthology and expression similarity

To establish the comparability of the bank vole genome to other Glires annotations, we first analyzed orthology in a broad phylogenetic context. OrthoFinder identified 20,547 orthogroups representing 97.9% of the genes across all 24 analyzed genomes (including the human outgroup). Of the orthogroups, 6,158 had all species present. In the draft *de novo* bank vole genome, there were 27,824 annotated genes, of which 84.2% were assigned to an orthogroup. Bank vole genes were present in 16,250 orthogroups. On average, the genomes included in the OrthoFinder analysis had 19,814 genes, with 98.2% of those assigned to orthogroups.

We also assessed differential gene expression between bank vole and mouse molars across early development, as mice are the mammalian model species in which dental development is most commonly studied. We focused on keystone dental gene categories established by Hallikas and co-authors [43] and collected data for the bank vole m1 at the same embryonic days (E13, E14, and E16) used in their study, during which the tooth crown is patterned [43]. Null mutations in keystone dental genes affect embryonic dental development; the effects of these genes were established based on literature reviews of in vivo experimental results [43]: “shape” genes cause morphological errors; “eruption” genes prevent tooth eruption; “progression” genes stop the developmental sequence; “tissue” genes cause defects in enamel and dentin; “developmental process” genes are annotated with the “GO:0032502” gene ontology term; “dispensable” genes, while dynamically expressed in developing teeth, have no documented effect on phenotype, but nevertheless may be important for tooth formation; and 11“double” genes, five pairs that function redundantly with a paralog and only produce a phenotype when both genes are mutated, and one gene that produces a phenotype when the paralog is heterozygous. Although more genes from the dispensable list may fit into this “double” category, they have not be experimentally confirmed [43]. The group “other” is composed of the remaining protein coding genes [43].

**Fig. 2:**
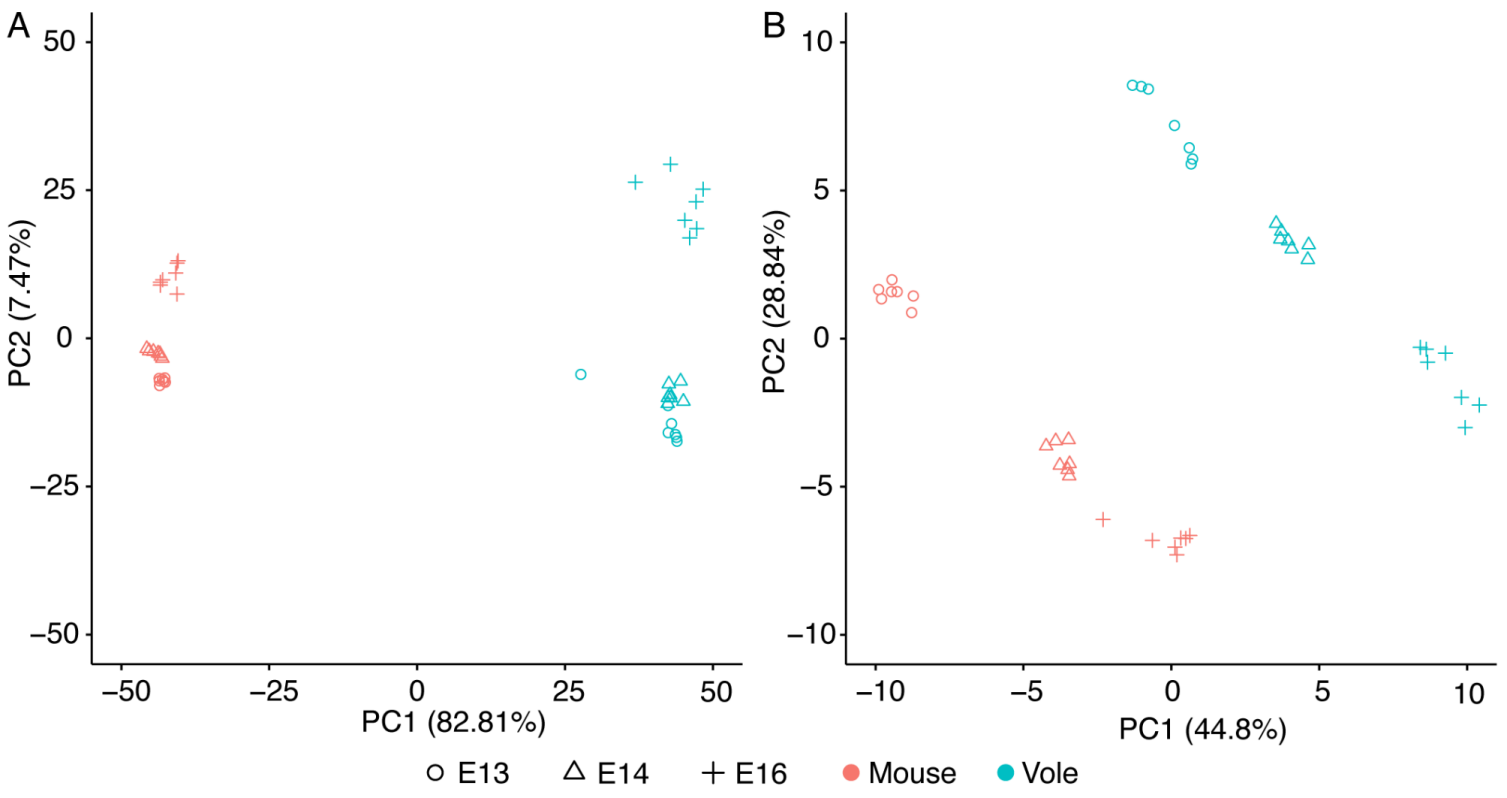
Principal component (PC) analyses of differentially expressed genes in mouse and bank vole m1. **A** PC1 and PC2 of the 500 most variable genes, showing a clear differentiation between species along PC1 and differentiation between age classes along PC2. **B** PC1 and PC2 of the keystone dental genes. Both PC1 and PC2 separate age classes within, but not between, the species, likely due to differences in developmental timing and molar morphology between mice and voles

Ordination of gene expression results from the bank vole and published mouse data [43] by principal component analysis showed a distinct separation between the mouse and bank vole along the first principal component (PC1) of the 500 most variable genes (Fig. 2A). PC1 explained 82.81% of the variance in these genes; there are distinct, species-specific expression patterns in these tissues. PC2 appears to distinguish E13 and E14 samples from E16 samples in both species. Ordination of the keystone dental genes showed two distinct, parallel trajectories for the mouse and bank vole (Fig. 2B). Within this focused set of genes, however, PC1 and PC2 explain less variance (44.8% and 28.84% respectively) and align less clearly to species and age.

**Fig. 3:**
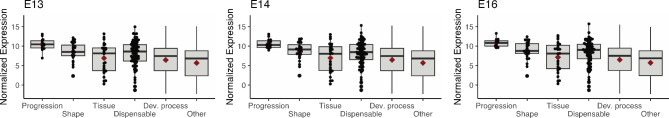
Box and whisker plots showing normalized log base 2 expression levels for each keystone gene category in bank vole m1 at embryonic days 13, 14, and 16. Horizontal bar and diamond within each box represent the median and mean values. Individual datapoints are displayed for smaller keystone gene categories. Gene expression profiles at these stages are comparable to mouse and rat molars at analogous developmental stages [43]

Developing bank vole molars at E13, E14, and E16 expressed keystone dental genes in overall proportions like those observed during mouse and rat molar development [[43], (Fig. 3)]. As with the mouse and rat [43], log counts for progression genes were most consistently upregulated compared to other keystone dental genes (permutation test p-values < 0.05 except for the E13 dispensable comparison; see Supplementary Material 1). Yet, we found individual genes involved in cusp patterning and morphology [44, 45] differed between the mouse and the vole: *Bmp2*, *Shh*, *p21* (also known as *Cdkn1a*), and *Msx2* were overexpressed in vole molars relative to mouse molars. This result agrees with the faster patterning and greater number of cusps in the vole molar [45]. *Fgf10*, which is associated with delayed root formation later in vole molar development [9], also was overexpressed in vole molars. Altogether, the draft bank vole genome assembly and annotation provide a reliable readout of vole tooth development and good bases to explore links between determinate and indeterminate growth in molars.

### Loss of synteny in dental orthogroups

**Fig. 4:**
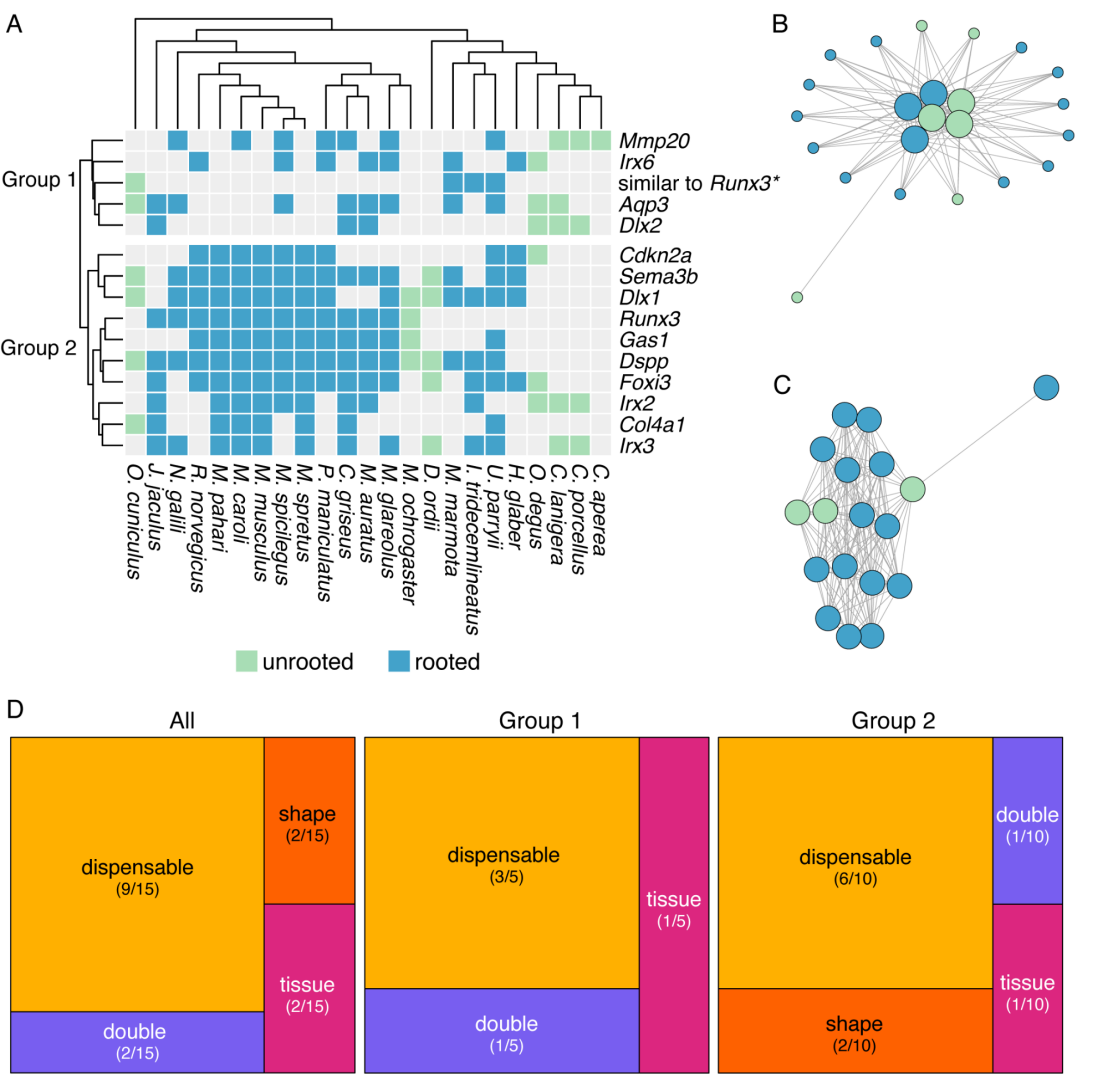
**A** Presence (colored boxes) or absence (gray boxes) of gene sequences for each species in hierarchical orthogroups where fewer than half of the species with unrooted molars had conserved synteny. Columns are ordered according to phylogenetic positions (top) and rows are ordered by Euclidean distance clustering. Rows are split into two major groups: group 1, in which synteny is not conserved across Glires, and group 2, in which synteny is not conserved mainly in species with unrooted molars. * = One hierarchical orthogroup represented only four gene sequences annotated based on similarity to *Runx3*. **B** An example of a synteny network for genes in Group 1, displayed using the Fruchterman-Reingold layout algorithm in the R package *iGraph* [46]. Small circles represent genes in the synteny network that are not part of the hierarchical orthogroup, large circles represent genes in the hierarchical orthogroup, and lines between circles represent a syntenic relationship between two species. Circle color represents whether species has rooted or unrooted molars following the same key in A. **C** An example synteny network for genes in Group 2, displayed using the Fruchterman-Reingold layout algorithm in the R package *iGraph* [46]. Circles represent genes in the hierarchical orthogroup, and lines between circles represent a syntenic relationship between two species. Circle color represents whether species has rooted or unrooted molars following the same key in A. **D** Treemaps representing the keystone gene categories for all hierarchical orthogroups in this figure, the Group 1 hierarchical orthogroups, and the Group 2 hierarchical orthogroups. Most genes in each group are in the dispensable keystone gene category, which includes genes that are dynamically expressed during dental development but have no documented effect on phenotypes

Next, to compare convergent changes in gene order between species with rooted and unrooted molars, we used the infomap clustering algorithm and produced 19,694 microsynteny clusters from the synteny blocks estimated by MCScanX. We did not expect dental genes to share the same microsynteny cluster, and instead examined whether each gene was in the same microsynteny cluster in species with rooted or unrooted molars. We identified 15 hierarchical orthogroups in which synteny was not conserved for at least half of the Glires with unrooted molars (Fig. 4). The genes form two groups (Fig. 4A), group 1, lacking synteny across Glires, and group 2, lacking synteny mainly in species with unrooted molars. Most of these genes also are missing from the orthogroups; only *Mmp20*, *Irx6*, *Aqp3*, *Sema3b*, and *Col4a1* were well represented in their orthogroups but not in their synteny networks (full comparisons of orthology and synteny are in Supplementary Material 2). Overall, these genes represent multiple categories of keystone dental genes. Most genes lacking conserved synteny in species with unrooted molars are in the “dispensable” category (Fig. 2D), thus the relationship between differences in these genes and tooth phenotypes is unclear, at least during embryonic development.

### Multiple dental genes under positive selection

At the level of individual genes, we hypothesized that dental genes are undergoing positive selection in species with unrooted molars. Positive selection analyses in PAML (phylogenetic analysis by maximum likelihood [47]) identified 6 dental gene orthogroups undergoing site-specific positive selection across Glires. Four orthogroups with site-specific positive selection also lacked synteny among Glires with unrooted molars: *Col4a1*, *Dspp*, *Runx3*, and the four-gene orthogroup with sequences similar to *Runx3* (Fig. 4A). We then assessed genes for site-specific positive selection in species with unrooted molars compared to species with rooted molars (branch-and-site-specific positive selection [48]), focusing on those genes with site-specific positive selection or evidence for loss of synteny. Two genes, *Dspp* and *Aqp1*, were undergoing this branch-and-site-specific positive selection. Both genes had a single highly supported site (posterior probability > 0.95) under positive selection in species with unrooted molars based on the Bayes Empirical Bayes method for identifying sites under selection implemented in PAML [49]. *Dspp* also had multiple sites with moderate support (posterior probability > 0.75). Selection patterns on each gene differed. Maximum likelihood estimates of selection for *Dspp* support a mixture of sites under purifying and neutral selection. Sites under positive selection in the species with unrooted molars (foreground branches) were evenly distributed among sites under both modes of selection in species with rooted molars (background branches). For *Aqp1*, nearly all sites were under purifying selection and a small proportion (7%) were under neutral selection. The few sites under positive selection in the foreground branches were mainly under purifying selection on background branches. The complete list of dental genes with hierarchical orthogroups, microsynteny clusters, and positive selection test results are available in Supplementary Material 2. In agreement with the positive selection analyses in PAML, analyses of the selection intensity (*k*) in RELAX did not support an interpretation of relaxed selection for either *Dspp* or *Aqp1*.

**Fig. 5:**
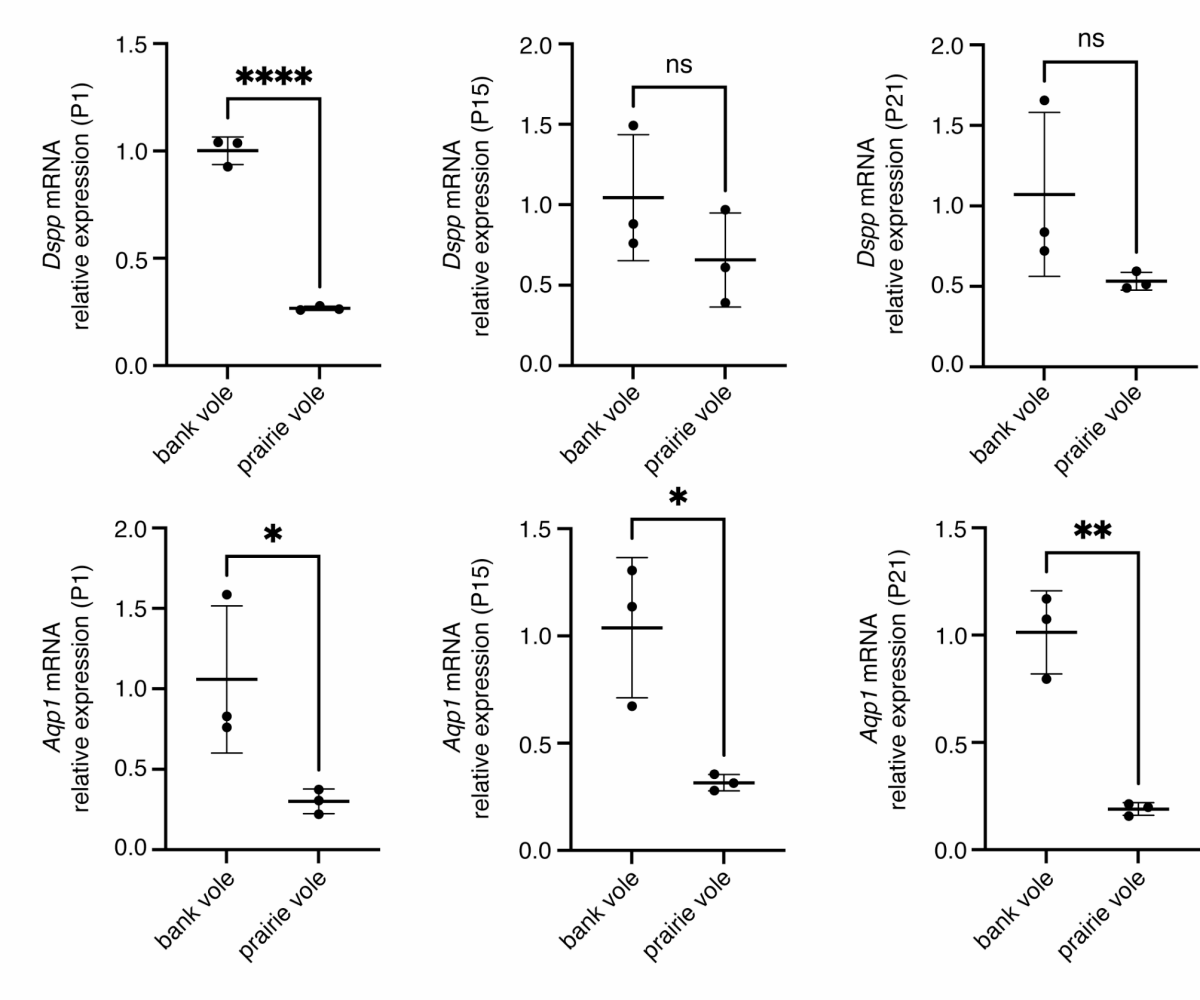
Quantitative PCR comparisons of *Dspp* and *Aqp1* expression between bank vole and prairie vole m1 at postnatal days 1, 15, and 21 (P1, P15, P21). Expression levels for both genes are lower in the prairie vole (unrooted molars), which supports the positive selection detected for these genes in species with unrooted molars. Each point represents an average of three technical replicates for a single biological replicate

Because genes under positive selection are often expressed at lower levels than genes under purifying selection [50–53], we also compared expression levels of *Dspp* and *Aqp1* in first lower molars (m1) at postnatal days 1, 15, and 21 (P1, P15, and P21) in bank voles (rooted molars) and prairie voles (unrooted molars) using quantitative PCR. These timepoints capture times before and after tissue changes that signify the onset of root formation in bank voles. Prairie vole molars expressed *Aqp1* at significantly lower levels than bank vole molars across all three ages (Fig. 5). Prairie vole P1 molars expressed significantly lower levels of *Dspp* than bank vole molars; at P15 and P21, their molars expressed *Dspp* at lower, but not statistically significantly different, levels than their bank vole equivalent.

### Few changes of secondary structure at positively selected sites

**Fig. 6:**
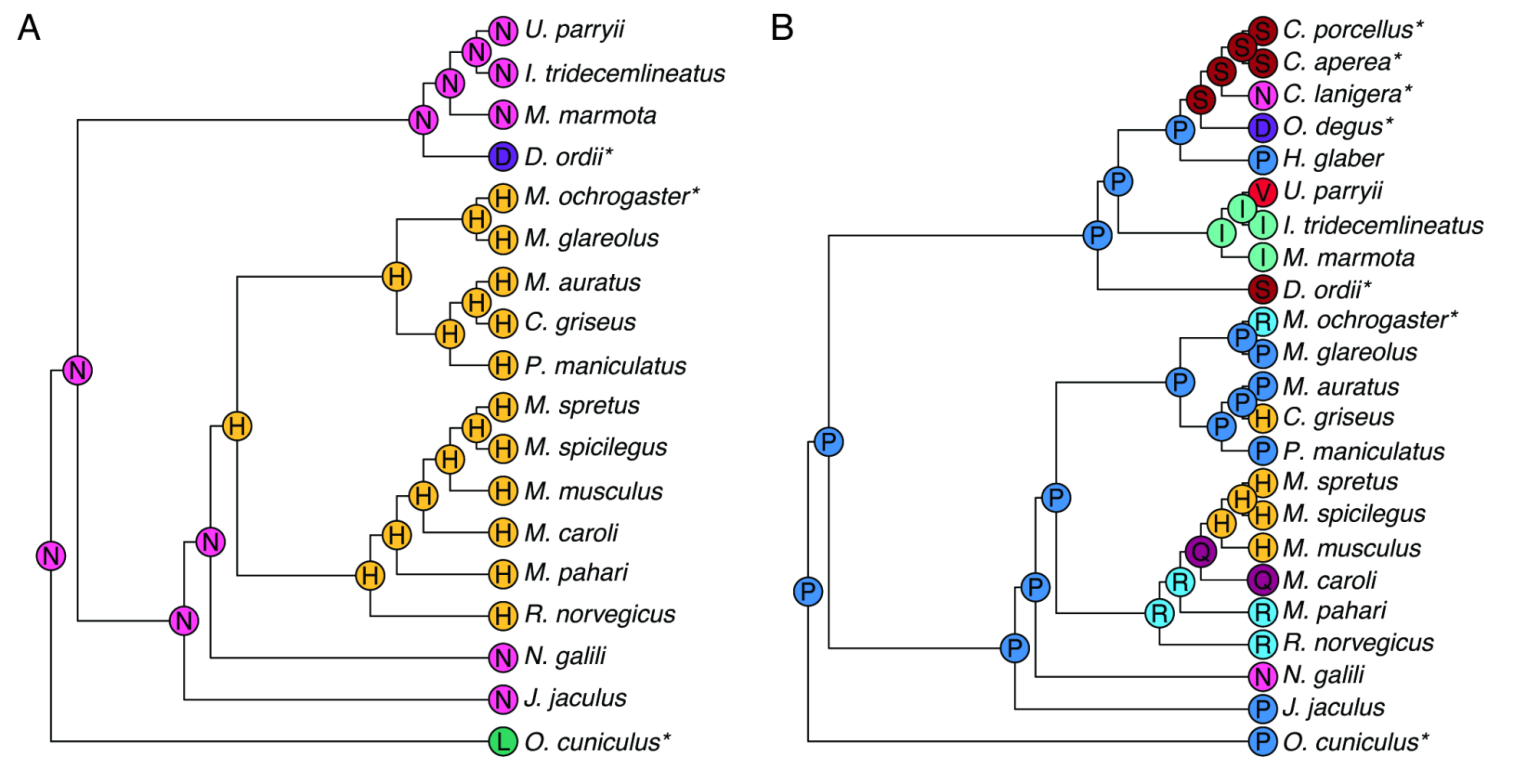
Ancestral state reconstructions of the residue under positive selection in PAML tests. Letters at tips and internal nodes represent IUPAC codes for amino acids and * denotes species with unrooted molars. **A*** Dspp*; **B*** Aqp1*

**Fig. 7:**
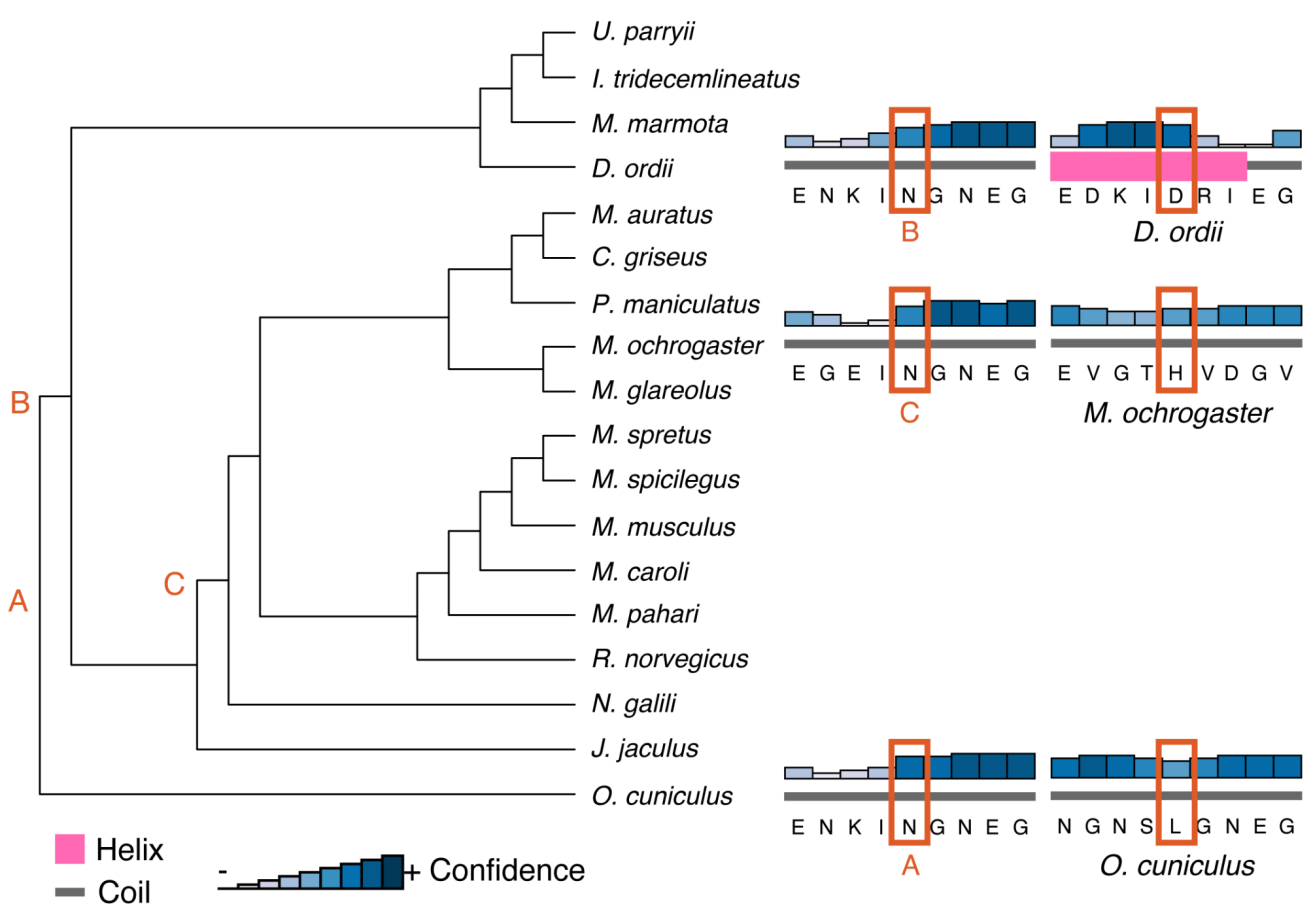
PSIPRED secondary structure predictions for the three species with unrooted molars represented in the *Dspp* sequences. Letters correspond to the most recent ancestor of each tip species where the amino acid at the site under positive selection differed: A, the predicted ancestor of *O. cuniculus*; B, the predicted ancestor of *D*. *ordii*; and C, the predicted ancestor of *M. ochrogaster*. Structure predictions, the relative confidence of the prediction, and the amino acid sequence for each pair of extant species and ancestor are on the right

Finally, to detect whether substitutions at sites under positive selection influenced protein structure and evolution, we analyzed ancestral states and secondary structure across Glires. We first reconstructed ancestral sequences along the internal nodes of the Glires phylogeny for the genes undergoing branch-and-site specific positive selection to assess potential secondary structural changes in their protein sequences. At the best-supported site in *Dspp* (position 209 in the gapped alignment, Supplementary Material 3), there were three major amino acid changes. Two substitutions were in species with unrooted molars only; *Oryctolagus cuniculus* had a leucine (N209L) at this position and *Dipodomys ordii* had an aspartic acid (N209D) at this position (Fig. 6A). All muroids (the clade including the voles in family Cricetidae and mice and rats in family Muridae) in our phylogeny substituted histidine (N209H) for the asparagine at this position. The secondary structure predicted at this position was a coil for most sequences but a helix for the *D. ordii* sequence (Fig. 7). *Aqp1* sequences varied greatly at the position under putative positive selection in species with unrooted molars (Fig. 6B, position 294 in the gapped alignment, Supplementary Material 4). These changes did not affect the predicted secondary structure of the protein near this residue, which was a coil for all sequences tested. All secondary structure predictions are available in Supplementary Material 5.

## Discussion

Convergent evolution of continuously-growing molars in Glires [1] presents a system to investigate molecular mechanisms of tooth root formation. If the same genomic changes appear in species with unrooted molars but not their relatives, it would support the relevance of those genes or genomic features for the maintenance of dental stem cells and formation of unrooted teeth. We sequenced the bank vole genome to support the development of a comparative system for studying tooth root development and tested the hypothesis that dental genes are undergoing positive selection in species with unrooted molars. We predicted that lack of conserved syntenic relationships in species with unrooted molars could place dental genes in regulatory and selective environments that promote changes among genes relevant to tooth root formation. Synteny comparisons identified 15 dental genes lacking conserved syntenic relationships across Glires and two dental genes, *Dspp* and *Aqp1*, under positive selection in species with unrooted molars. These genes may underlie the genomic changes required to maintain dental stem cells in the formation of hypselodont molars.

RNA sequencing of embryonic bank vole molars supported comparability of this system to mouse molars for studying dental development. Although molar morphology differs considerably across mammals, candidate-gene approaches have identified numerous conserved genes involved in tooth development and morphological patterning [54]. Studies of single genes or gene families have identified shape-specifying roles common to multiple species [45, 55–57], and high-throughput sequencing of mouse and rat molars demonstrate that both species express sets of dental development genes in similar proportions during early stages of tooth development [43]. The similarity of bank vole high-throughput RNA sequencing results (Fig. 3) to the mouse and rat results from previous studies [43] suggest overall expression patterns of keystone dental development genes within each stage are conserved across Glires.

Principal component analyses and differential expression analyses measuring changes between mouse and bank vole molars, however, showed that several dental genes’ expression levels differed significantly by species and age. Organ expression patterns can be conserved across species early in development but diverge over time, with some major organs displaying heterochronic shifts in some species [58]. If the major source of variation in dental gene expression patterns between mice and bank vole molars were solely attributable to species, we might expect to see clear separation between the species along the first or second principal component (PC1 or PC2), like that observed in PC1 of the 500 most variable genes (Fig. 2). Alternatively, if molar development follows the diverging expression patterns observed in other organs, we might expect just the earliest age classes to align on one, or multiple, PCs. Instead, we found two trajectories that were nearly parallel across PC1 and PC2 and multiple genes that were significantly differentially expressed with respect to species and age. This variation between species is likely driven by the larger number of cusps in the vole molar, and corresponding upregulation of genes regulating cusp formation, further highlighting the need to establish a morphologically similar molar model to isolate the changes related to root formation.

We identified 15 genes which were not syntenic in at least half of the species with unrooted molars, and six genes undergoing site-specific positive selection across all Glires. Four of the orthogroups with site-specific positive selection lacked synteny in species with unrooted molars, yet only *Col4a1* was well represented among these species in its orthogroup. Although we predicted loss of synteny for dental genes in Glires with unrooted molars could result in sequence evolution by placing genes in new selective contexts, we did not find many non-syntenic dental genes under branch-and-site-specific positive selection. The two genes undergoing branch-and-site-specific positive selection in species with unrooted molars, *Dspp* and *Aqp1*, both maintained their synteny relationships across the Glires studied. Maximum likelihood estimates of selection on each site for the genes with branch-specific positive selection revealed different overall selective pressures on *Dspp* and *Aqp1*; *Dspp* sites on background branches (i.e., branches with species that have rooted molars) were under a mix of purifying and neutral selection, while nearly all *Aqp1* background branch sites were under purifying selection. These selection regimes suggest there is greater conservation for *Aqp1* function across Glires than for *Dspp* function. Gene duplication can result in functional redundancy and evolution toward a novel function in some genes [59–62], which may explain positive selection in *Aqp1*, as there are other aquaporin family genes present. Although *Dspp* has no paralogs, it overlaps functionally with other SIBLING family proteins (e.g., *Opn*, *Dmp1*) [63, 64].

*Aqp1* and *Dspp* play different functional roles during dental development. Under the keystone dental development gene framework, *Aqp1* is a “dispensable” gene: developing teeth express it, but tooth phenotypes do not change in its absence. *Aqp1* is expressed in endothelia of microvessels in the developing tooth [65, 66]. *Dspp* may be particularly relevant for the formation of an unrooted phenotype if its expression domain or function have been modified in species with unrooted molars. *Dspp* is a “tissue” category keystone dental gene, meaning the main effects of a null mutation occur during the tissue differentiation stage of dental development, particularly in the formation of enamel and dentin [43]. Null mutations of *Dspp* cause dentin defects in a condition called dentinogenesis imperfecta [67, 68]; in some patients, teeth form short, brittle roots [68, 69]. *Dspp* knockout mice also exhibit the shortened root phenotype, among a variety of other defects in both endochondral and intramembranous bone, due to the disruption of collagen and bone mineralization [70–72].

Ancestral sequence reconstructions and estimated secondary protein structures allowed us to assess whether nonsynonymous substitutions at sites under positive selection resulted in structural differences, thus potentially affecting protein function. Although unrooted molars are a convergent phenotype across Glires, the sites under positive selection did not converge on the same amino acid substitution in species with unrooted molars, and *Aqp1* appeared particularly labile at this residue. The non-synonymous substitutions at these sites often resulted in changes of properties of the amino acid in the sequence, for example in *Dspp*, polar asparagine was replaced with non-polar leucine in *O. cuniculus*. Only one of these substitutions changed the predicted secondary structure. Nevertheless, single amino acid substitutions do produce dental phenotypes for both *Dspp* [73] and *Aqp1* [74], thus we cannot rule out functional changes in these genes in species with unrooted molars.

Although the exact relationship between gene expression and sequence divergence remains unclear [75], studies of genome evolution across small numbers of mammal species show correlations between gene sequence divergence and levels of expression [76]. In particular, highly-expressed genes are more likely to experience purifying selection [50–53], while lowly-expressed genes and tissue-specific genes may experience positive selection [51]. The decreased expression of *Dspp* and *Aqp1* in prairie vole m1 compared to that of the bank vole m1 thus supports our finding of positive selection in these genes in species with unrooted molars. If all species with unrooted molars also exhibit decreased expression levels of *Dspp* and *Aqp1*, it could suggest a strong link between lower levels of the genes and the unrooted phenotype.

Without analyses of functional variation caused by positive selection at these coding sites, or spatial sampling to determine where these genes may be expressed during development, we are limited from exploring the specific effects of *Dspp* and *Aqp1* on root formation. That expression of both genes is lower (but not significantly different for *Dspp*) between bank and prairie voles after the onset of molar root formation prior to P15 further underscores the complexity of root formation genetics. Nevertheless, we found evidence for evolution of these genes in Glires with unrooted molars, and *Dspp* especially has clinical relevance for tooth root formation. Future studies should explore the spatial distribution of *Dspp* expression, which could be relevant to functional changes in Glires with unrooted molars. If positive selection and corresponding amino acid changes identified in *Dspp* here modify its expression domain or its interaction with yet-unidentified root formation co-factors, it may serially reproduce the unrooted incisor phenotype in molars.

Our analyses were limited by the small number of rodent species with sufficiently annotated genomes to be included in synteny and positive selection analyses. This lack of Glires with well-annotated, contiguous genomes resulted in a small phylogeny for ancestral state reconstructions, which thus did not encompass the full diversity of Glires tooth roots, and potentially weakened model-based genomic analyses. Although positive selection analyses using the Bayes Empirical Bayes criterion are robust to smaller sample sizes [49], incomplete sampling can affect estimations of ancestral characteristics [77]. Likewise, orthology inference with OrthoFinder relied on Glires genomes and annotations that may not be complete or correct for every protein. Although OrthoFinder assigned an average of 98.2% of genes from each genome to orthogroups, we nevertheless may have missed genes that could have been included in downstream analyses. Innovations in paleoproteomics also offer the opportunity to compare fossil species’ dental gene sequences directly to living and estimated ancestral sequences [78, 79]. By incorporating data for extinct Glires in future morphological and molecular analyses, we can further elucidate links between dental gene evolution and unrooted teeth.

## Conclusions

Analyses of the high-quality draft bank vole genome showed that bank vole early tooth development is comparable to other commonly used rodent models in dental development research. We identified 6 dental gene orthogroups that were undergoing site-specific positive selection across Glires and two genes, *Dspp* and *Aqp1*, that were undergoing site-specific positive selection in Glires with unrooted molars. *Dspp* appears particularly relevant to root formation, as loss-of-function mutations cause a dentin production defect that can result in shortened tooth roots. Future research must explore the functional role that *Dspp* plays in tooth root formation in Glires and other clades. The rodent dentary is an exciting system for understanding tooth development; it provides an easily manipulated set of tissues that can be produced quickly and features a lifelong population of stem cells in the incisor with genomic mechanisms that are potentially replicated across other teeth in species with unrooted molars. Our results identify candidate genes for future analyses, and the draft bank vole genome and annotation improve the utility of this species for comparative dental research that can uncover the genetic mechanisms of tooth root formation.

## Methods

### Tissue collection and sequencing

To assemble the bank vole genome, we sequenced tissues from a single adult male specimen housed in a colony at the UCSF Mission Center Animal Facility. We euthanized the animal according to UCSF IACUC protocol AN189916 and harvested muscle, kidney, heart, and liver tissue, which were immediately frozen at -80 °C. Tissues were sent to a third-party sequencing service, where they were combined and homogenized to achieve appropriate mass for high molecular weight DNA extraction. We targeted 60x coverage with 150 base pair (bp) reads using 10X Chromium linked-read chemistry [80, 81] sequenced on the Illumina platform. We also targeted 10x coverage with Pacific Biosciences SMRT long-read chemistry. For genome annotation and gene expression analyses, we collected seven biological replicates each of first lower molars at embryonic days 13–16 (E13, E14, E15, E16), second lower molars at E16, and jaw tissues at E14 under University of Helsinki protocols KEK16-021, KEK19-019, and KEK17-030 and stored them in RNAlater at -80 °C for RNA sequencing, following a tissue harvesting protocol established for mice and rats [43]. We extracted RNA from these tissues using a guanidium thiocyanate and phenol-chloroform protocol combined with an RNeasy column purification kit (Qiagen) based on the keystone dental gene protocol [43]. Single-end 84 bp RNA sequencing was performed using the Illumina NextSeq 500 platform.

### Genome assembly and quality control

We first assembled only the 10X Chromium linked reads using the default settings in Supernova 2.1.1 [80, 81]. We selected the “pseudohaplotype” (pseudohap) output format, which randomly selects between potential alleles when there are two possible contigs assembled for the same region. This option produces two assemblies, each with a single resolved length of the genome sequence [80–82]. We used the lower-coverage, long-read data for gap filling and additional scaffolding. First, we estimated the genome’s length using the raw sequence data in GenomeScope [83], which predicted a length of 2.6 gigabases. We then performed error correction of the long reads using Canu [84], removing reads shorter than 500 bp and disregarding overlaps between reads shorter than 350 bp. We kept only those reads with minimum coverage of 3x for scaffolding. Following long read error correction, we used Cobbler and RAILS [85] with a minimum alignment length of 200 bases to accept matches for gap filling and scaffolding of both pseudohap assemblies.

**Fig. 8:**
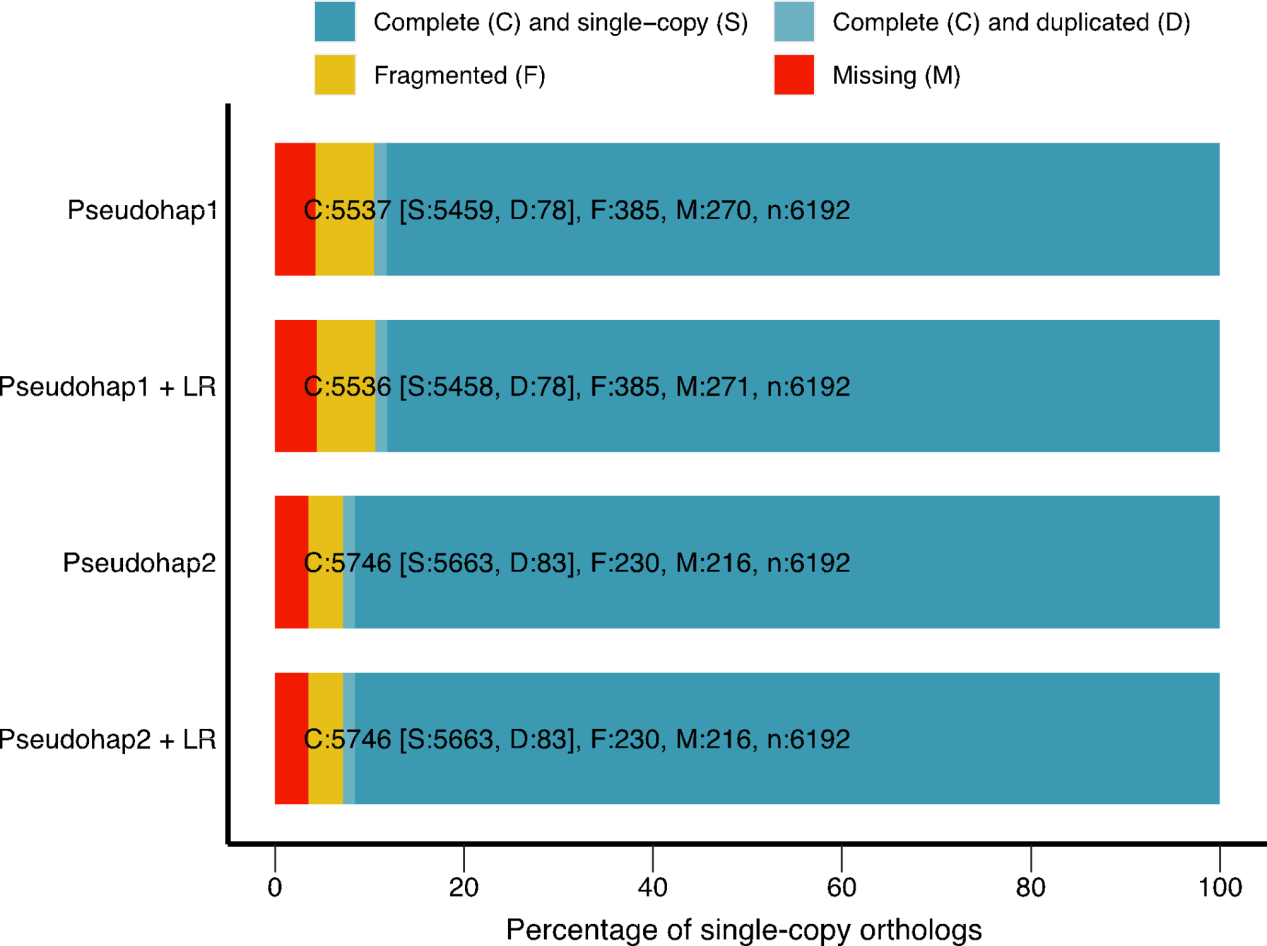
BUSCO single-copy ortholog recovery for each “pseudohaploid” version of the draft bank vole genome assembly and the version after long-read scaffolding (denoted by “+ LR”). Each bar represents the cumulative proportion of the 6,192 single-copy orthologs for Euarchontoglires identified by BUSCO represented by complete single-copy, complete-duplicated, fragmented, and missing orthologs. The Pseudohap2 and Pseudohap2 + LR assemblies had the best single-copy ortholog recovery

For quality control, we assessed both unscaffolded and long-read scaffolded pseudohap assemblies by standard assembly length statistics with QUAST [86] and presence of single-copy orthologs with BUSCO v3 [87]. Both scaffolded assemblies were approximately 2.44 Gigabases long, with an N50 (the length of the shortest scaffold at 50% of the total assembly length) of 4.6 Megabases; we refer to them as Pseudohap1 + LR and Pseudohap2 + LR. The Pseudohap1 + LR assembly had 17,528 scaffolds over 1000 bp long, and the Pseudohap2 + LR assembly had 17,518 scaffolds over 1000 bp long (Table 1). BUSCO searched for universal single-copy orthologs shared by Euarchontoglires, recovering 89.4% of these genes in the scaffolded Pseudohap1 + LR assembly and 92.8% of the single-copy orthologs in the scaffolded Pseudohap2 + LR assembly (Fig. 8). The two assemblies were similar length and contiguity, but we based annotation and downstream analyses on Pseudohap2 + LR because it recovered more single-copy orthologs.

**Table 1:** QUAST assembly statistics for *de novo* bank vole (*Myodes glareolus*) genome assemblies

	Pseudohap1	Pseudohap1 + LR	Pseudohap2	Pseudohap2 + LR*
Largest contig	27,939,478	32,658,832	27,937,749	32,657,565
Total length	2,434,151,515	2,441,426,554	2,434,099,357	2,441,472,313
GC (%)	41.88	41.89	41.88	41.89
N50	4,187,179	4,579,815	4,187,179	4,558,134
N75	1,689,669	1,818,134	1,687,188	1,810,460
L50	170	153	170	154
L75	388	357	388	358
Ns per 100 kbp	1151.99	1030.75	1151.96	1030.48

*assembly used for annotation and downstream analyses in this paper

### Genome annotation

We annotated the genome using multiple lines of evidence in three rounds of the MAKER pipeline [88–90]. For evidence from gene transcripts, we assembled a *de novo* transcriptome assembly of the single-end RNA sequences pooled from all molar and jaw tissues using Trinity [91]. We also included cDNA sequences from the *Mus musculus* assembly GRCm38 to provide additional transcript evidence from a close relative with a deeply annotated genome. We used SwissProt’s curated protein database to identify protein homology in the genome. Two libraries of repeats provided information for repeat masking: the Dfam Rodentia repeat library [92–94] and a custom library specific to the bank vole estimated with a protocol modified from Campbell et al. [89]. The custom library features miniature inverted-repeat transposable elements identified with default settings in MiteFinder [95], long terminal repeat retrotransposons extracted with the GenomeTools LTRharvest and LTRdigest functions [96] based on the eukaryotic genomic tRNA database, and *de novo* repeats identified with RepeatModeler [97]. We combined elements identified by these programs into a single repeat library, then removed any elements that matched to a custom SwissProt curated protein database excluding known transposons. The custom repeat library is available in Supplementary Material 6. We trained a custom gene prediction model for MAKER as well. The first iteration of the model came from BUSCO’s implementation of augustus [98]. Between each round of MAKER annotation, we further updated the gene prediction model with augustus.

MAKER considered only contigs between 10,000 and 300,000 bp long during annotation. The second and third iterations of MAKER used the same settings but excluded the “Est2genome” and “protein2genome” functions, as recommended in the MAKER tutorial. We included a SNAP [99] gene prediction model based on the output of the first round of annotation during the second and third iterations of MAKER annotation. Annotation quality (i.e., agreement between different lines of evidence and the MAKER annotation) was assessed visually in JBrowse after each iteration and using *compare_annotations_3.2.pl* [100], which calculates the number of coding and non-coding sequences in the annotation in addition to basic statistics about sequence lengths. The MAKER annotation covered 2.41 Gb of the scaffolded Pseudohap2 assembly in 4,125 scaffolds. These scaffolds contained 27,824 coding genes (mRNA) and 15,320 non-coding RNA sequences. The average gene length was 12,705 bp. Most annotations (91.4%) had an annotation edit distance (AED) of 0.5 or better. AED is a measure of congruency between the different types of evidence for an annotation, where scores closer to zero represent better-annotated genes [101].

### Orthology and comparability to glires and mice

We analyzed orthology and synteny of the bank vole genome to understand gene and genome evolution related to dental development across Glires with rooted and unrooted molars. We obtained genomes from Ensembl for 23 Glires species and one phylogenetic outgoup, *Homo sapiens* (Table 2). These genomes all had an N50 over 1 Mb, which improves synteny assessment [102]. We first analyzed all 24 genomes for groups of orthologous genes (orthogroups) in OrthoFinder [103], providing a tree topology based on the Ensembl Compara reference tree (Fig. 1) to guide orthology detection. Because we would not analyze the human outgroup in downstream analyses, we implemented the OrthoFinder option that splits orthogroups at the root of Glires (hierarchical orthogroups), thus any group of orthologs studied here represents only genes with shared, orthologous evolutionary history within Glires. We selected MAFFT [104] for multiple sequence alignment and fastme [105] for phylogenetic tree searches within OrthoFinder. We retained the gene trees estimated for each orthogroup for downstream analyses.

**Table 2:** Genomes used in orthology, synteny, and positive selection analyses

Species	Assembly	Citation
*Myodes glareolus*	CUNY_Mgla_1.0	This paper
Cavia porcellus*	Cavpor3.0	[106]
Cavia aperea*	CavAp1.0	[107]
*Marmota marmota*	marMar2.1	[108]
Microtus ochrogaster*	MicOch1.0	[109]
*Mus musculus*	GRCm39	[110]
Oryctolagus cuniculus*	OryCun2.0	[106]
Dipodomys ordii*	Dord_2.0	[106]
*Jaculus jaculus*	JacJac1.0	[111]
*Rattus norvegicus*	Rnor_6.0	[112]
*Mus pahari*	PAHARI_EIJ_v1.1	[113]
*Mus caroli*	CAROLI_EIJ_v1.1	[113]
*Mus spretus*	SPRET_EiJ_v1	[114]
*Mus spicilegus*	MUSP714	[115]
*Cricetulus griseus*	CHOK1GS	[116]
*Mesocricetus auratus*	MesAur1.0	[117]
*Peromyscus maniculatus*	HU_Pman_2.1	[118]
*Nannospalax galili*	S.galili_v1.0	[119]
Octodon degus*	OctDeg1.0	[120]
*Heterocephalus glaber (F)*	HetGla_female_1.0	[121]
Chinchilla lanigera*	ChiLan1.0	[122]
*Urocitellus parryi*	ASM342692v1	[123]
*Ictidomys tridecemlineatus*	SpeTri2.0	[124]
Homo sapiens**	GRCh38	[125]

*Species with unrooted molars; **Peptide annotation used as outgroup only in OrthoFinder analysis

To assess the comparability of the bank vole molar to mouse molars, we performed RNA sequencing of bank vole molars modeled on published analyses of mouse molars [43]. We performed quality control and filtering of the short reads for the seven replicates of bank vole first molar tissues at E13, E14, and E16 using the nf-core/rnaseq v. 3.11.2 workflow [126]. RNA sequencing reads were evaluated and adapter sequences were filtered using FastQC v. 0.11.9 [127] and Cutadapt v. 3.4 [128], and ribosomal RNA was removed using SortMeRNA v. 4.3.4 [129]. We then aligned trimmed sequences against the draft bank vole annotation using Salmon v. 1.10.1 [130]. Counts were then normalized by gene length. We categorized gene count data into functional groups based on their established roles in tooth bud development [43] using the one-to-one orthology list between the draft bank vole genome and the mouse GRCm39.103 genome annotation generated by OrthoFinder. Using the rlog function of DESeq2 [131], we normalized gene counts within each functional group on a log_2_ scale. A permutation test assessed whether the mean counts of the progression, shape, and double functional groups were significantly different from genes in the tissue, dispensable, and “other” groups (which are potentially relevant later in development) based on 10,000 resampling replicates of the dataset [43].

We also assessed differential expression between the bank vole first molar and published mouse m1 data at the same three time points (GEO accession GSE142199 [43]), combining the data based on the one-to-one orthology relationships used in the functional permutation analysis. Using the mouse E13 molar as the reference level, we modeled expression as a response to species (mouse or vole), embryonic day (E13, E14, or E16), and the interaction between species and day. We considered as significant any gene with a log fold change greater than 1, log fold change standard error less than 0.5, and false discovery rate adjusted p value less than 0.05.

### Synteny and positive selection analyses

Convergence on unrooted molars across Glires may suggest shared mechanisms underlying this morphology. Although dental development genes are spread throughout the genome, we assessed whether each gene remained in the same local arrangement across species of Glires, or if losses of synteny reflected the acquisition of unrooted molars. We prepared each genome annotation and sequence file for synteny analysis using the reformatting functions of Synima [132] to extract each peptide sequence associated with a gene coding sequence in the Ensembl annotation. Collinear synteny blocks estimated by MCScanX, which incorporates relative gene distance as a measure of gene density [133], formed the basis for synteny network analyses using the SynNet pipeline [134–136]. We inferred networks from the top five hits for each gene, requiring any network to have a minimum of 5 collinear genes and no more than 15 genes between a collinear block, settings that perform well for analyzing mammal genomes [136]. Using the infomap algorithm, we clustered the synteny blocks into microsynteny networks, from which we extracted network clusters corresponding to the list of keystone dental genes [43]. For each dental gene hierarchical orthogroup, we assessed whether genes of species with unrooted molars were missing from the synteny networks that contained other Glires species’ sequences, representing loss of synteny for those species.

To identify whether convergence on unrooted molars was related to differences in dental gene evolution, we performed positive selection analyses. We first aligned protein sequences for each dental gene orthogroup with clustal omega [137] using default settings. Based on universal translation tables, we obtained codon-based nucleotide alignments with pal2nal [138], removing sites in which any species had an indel (resulting in “ungapped” alignments) and formatting the output for analysis in PAML [47]. We pruned and unrooted the orthogroup gene trees from OrthoFinder to contain only tips representing the genes in each synteny network or orthogroup under analysis in PAML. We tested whether any of the genes were undergoing positive selection using a likelihood ratio test comparing site-specific models of “nearly neutral” and positive selection. In these models, ω, the ratio of nonsynonymous to synonymous nucleotide substitutions (also known as dN/dS), can vary at each codon site. In the “nearly neutral” model, ω can take values between 0 and 1, while the positive selection model allows sites to assume ω values greater than 1 [49, 139]. We estimated κ (the ratio of transitions to transversions) and ω from initial values of 1 and 0.5, respectively, for both tests.

Dental genes with significant site-specific positive selection or those lacking synteny in species with unrooted molars underwent positive selection analyses using the branch-and-site model of positive selection. This model allows ω to vary not only among codon sites, but also between “foreground” and “background” lineages [49]. We marked the species with unrooted molars as foreground lineages, then ran the model twice: once with ω unconstrained to detect sites undergoing positive selection only on foreground branches, and a second time and with ω fixed to 1, or neutral selection. This is an explicit test of positive selection developed to guard against identifying sites under relaxed selection as significant [48, 49]. A likelihood ratio test of the two models determined whether the lineage-specific positive selection model was more likely than a neutral model, and Bayes Empirical Bayes analyses [49] produced posterior probabilities to identify sites under positive selection. To further ensure genes identified in this process were under positive selection, we assessed the selection intensity parameter *k* in the RELAX model [140]. We set the same foreground and background branches as in the PAML analyses, initialized each run using 25 random starting points on a 2000 × 2000 grid of rates and likelihoods, and repeated each run 10 times to check consistency across runs.

Genes under positive selection also tend to have lower expression levels [51], thus we compared expression of the genes with branch-and-site specific positive selection between the prairie (unrooted molars) and the bank vole (rooted molars) to provide further support for selective differences. We collected three biological replicates of first lower molars from both species at three postnatal stages (P1, P15, and P21) and immediately preserved them at -80 °C in lysis buffer (Buffer RLT; Qiagen) supplemented with 40 µM dithiothreitol. These postnatal timepoints bridge the onset of root formation in bank voles, to further assess the effects of these genes may have on dental development. RNA was extracted from homogenized tissues using a RNeasy column purification kit (Qiagen). We assessed concentration and purity of extracted RNA using a NanoDrop 2000 spectrophotometer (ThermoFisher Scientific). Using 1 µg of RNA, we synthesized cDNA using a high-capacity cDNA reverse transcription kit (ThermoFisher Scientific). We used 1 µL diluted cDNA (1:3 in ddH_2_O) and iTaq Universal SYBR Green Supermix (Bio-rad) in the Bio-rad CFX96 real-time PCR detection system for qPCR experiments. Each biological replicate was sequenced three times and the resulting measurements were averaged across the three technical replicates. We normalized cycle threshold values of genes of interest to GAPDH expression levels [141] and calculated relative expression levels as 2^−ΔΔCT^. A two-tailed unpaired t-test calculated in Prism 9 measured whether expression of these genes significantly differed between bank voles and prairie voles. The oligonucleotide primers for each species and gene are in Supplementary Material 7.

### Sequence and secondary structure evolution

We performed ancestral sequence reconstruction on the codon sequences of the genes that had evidence of branch-and-site specific positive selection to understand how the sequence has changed through time. The gapped clustal omega alignments were the basis for ancestral sequence reconstruction on the Glires species tree (Fig. 1) using pagan2 [142]. For each gene, we plotted amino acid substitutions at the site with potential positive selection. Finally, we predicted secondary structures (i.e., helices, beta sheets, and coils) for each unrooted species’ protein sequence and the reconstructed ancestral sequence prior to the change at the site under positive selection using the PSIPRED 4.0 protein analysis workbench [143, 144]. Comparing these predictions across the phylogeny, we assessed how these substitutions at the site under selection may affect the structure of each protein.

## Electronic supplementary material

Below is the link to the electronic supplementary material.

Supplementary Material 1: Permutation test p-values

Supplementary Material 2: Orthology synteny and positive selection test results for all dental genes assessed

Supplementary Material 3: Gapped codon-based alignment for Dspp in fasta formatted sequences

Supplementary Material 4: Gapped codon-based alignment for Aqp1 in fasta formatted sequences

Supplementary Material 5: PSIPRED secondary structure predictions

Supplementary Material 6: Custom repeat library

Supplementary Material 7: Oligonucleotide primers

## Data Availability

The datasets supporting the conclusions of this article are available in the GenBank repository under the BioProject PRJNA1050237 (genome accession number JBBHLL000000000), Gene Expression Omnibus accession GSE250184, and in the article’s electronic supplementary material.
