# Supplementary material for: Bank vole genomics links determinate and indeterminate growth of teeth
Source: BMC Genomics. 2024 Oct 30;25:1000. doi: 10.1186/s12864-024-10901-2 (PMC11523675; doi:10.1186/s12864-024-10901-2)
Supplement: Supplementary file 7 — Supplementary Material 7: Oligonucleotide primers [file 12864_2024_10901_MOESM7_ESM.pdf]

Supplementary Material 7: Oligonucleotide primers

Primer sequences used for qPCR analyses of expression in bank vole and prairie vole M1 at three embryonic days.

Bank vole (*Myodes glareolus*, rooted molars)
Aqp1_forward: GGGCATTGAGATCATCGGCA
Aqp1_reverse: CCAGTGTAGTCAATCGCCAG
Dspp_forward: AGGAACTCCAGCACAGAATGA
Dspp_reverse: TCGTCCCTCCTACGTCTGTT
GAPDH_forward: GTGGGCAAAGTCATCCCAGA
GAPDH_reverse: GTGTAGCCCTTGATGCCCTT

Prairie vole (*Microtus ochrogaster*, unrooted molars)
Aqp1_forward: GCTCCTGCTCAGTTGTCAGAT
Aqp1_reverse: CACACCTCGAGCCAGGTCATT
Dspp_forward: GGAACTCCAGCACAGGATGA
Dspp_reverse: TCGTCCCTCCTACGTCTGTT
GAPDH_forward: TGGAGACAGCCGCTTCTTTT
GAPDH_reverse: GCGTCCAATACGGCCAAATC
